# New *In Situ*-Generated Polymer-Iodine Complexes with Broad-Spectrum Antimicrobial Activity

**DOI:** 10.1128/spectrum.00550-22

**Published:** 2022-09-20

**Authors:** Marina López-Álvarez, Herb Ulmer, Nico Klay, Jan Maarten van Dijl

**Affiliations:** a Department of Medical Microbiology, University of Groningen, University Medical Center Groningen, Groningen, The Netherlands; b X-Infex B.V., Bussum, The Netherlands; Shenzhen Bay Laboratory

**Keywords:** povidone-iodine, polyamide, polyurethane, bacteria, fungi, yeast

## Abstract

Iodine-containing systems show broad antiseptic properties that can be an invaluable tool in controlling infections in humans and animals. Here, we describe the first proof-of-concept studies on biocidal active polyamide- and polyurethane-iodine complexes that are produced *in situ* directly during the fabrication and/or polymerization process at laboratory and commercially relevant scales. These polymer-iodine materials are active against a broad range of microorganisms, including bacteria and fungi. It is suggested that the ease of manufacture and subsequent commercialization make said systems especially suited for applications as base materials for medical devices to reduce infection risks and control the spread of pathogens.

**IMPORTANCE** Infectious diseases are of mounting medical and public concern. A major contributor to this trend is the proliferation of medical implants, which are inherently vulnerable to microbial contamination and the subsequent onset of hospital-acquired infections. Moreover, implant-associated infections in humans are often difficult to diagnose and treat and are associated with substantial health care costs. Here, we present the development of biocidal active polyamide- and polyurethane-iodine complexes that are generated *in situ* during fabrication. We show that the excellent antiseptic properties of water-soluble povidone-iodine can be similarly realized in water-insoluble engineering plastics, specifically polyamide- and polyurethane-iodine. These complexes have inherent biocidal activity against major pathogenic bacteria and fungi.

## INTRODUCTION

Health care-acquired infections (HAIs) continue to be a source of countless unneeded deaths and excessive hospital costs. The main sources of HAIs are pathogenic microorganisms that have entered the body due to contamination and infection or to imbalances in the normal microbiota (i.e., dysbiosis) as a consequence of specific treatment conditions or weakened immune defenses. Often, an HAI forms at the site of a medical device (e.g., a central line catheter), which causes the introduction of unwanted microorganisms at a specific body site or becomes the scaffold for unwanted adhesion of bacteria already carried by the patient. In fact, medical devices are a significant source of HAIs and represent a serious concern in many clinical settings (1–3). Microbial infections caused by the use of medical devices such as sutures, catheters, central lines, and prostheses result in secondary infections that are often difficult to eradicate and compromise the well-being of the patient. Medical devices provide a perfect nidus for microbial adherence and the subsequent formation of biofilms, which are very difficult to eradicate. For these reasons, there is an urgent need to develop and evaluate new materials with biocidal properties in order to reduce the risk of medical device contamination and microbial attachment to said medical devices.

The antiseptic properties of iodine have been known for more than 150 years, but its use as an infection control substance was not fully realized until the development of iodophors. An iodophor is a preparation in which the water-insoluble iodine is complexed with a solubilizing agent to generate a stable water-soluble material capable of releasing free iodine in solution. The solubilizing agents are generally surfactants and/or water soluble polymers, with multiple water-soluble iodophors developed to prevent infections (4). The water-soluble iodophor complex polyvinylpyrrolidone-iodine, also known as povidone-iodine (PVP-I), is a potent broad-spectrum antimicrobial active with excellent antiseptic properties against bacteria, including methicillin-resistant Staphylococcus aureus (MRSA) and mycobacteria (5), fungi (6), protozoa (7), and both enveloped and nonenveloped viruses, such as the poliovirus, immunodeficiency viruses (7, 8), influenza viruses (9, 10), and the coronaviruses severe acute respiratory syndrome (SARS) (11), Middle East respiratory syndrome (MERS) (12), and SARS coronavirus 2 (SARS-CoV-2) (13). Topical formulations of PVP-I have been widely used over the years for disinfection, wound care, and treatment of burns (9), and despite its common use for more than 60 years, no microbial resistance to PVP-I has ever been reported (6). However, the PVP-I complex is water soluble, and thus, its applications in health care are generally limited to surface and skin disinfection to minimize the risk of infections.

The research question we addressed in the present study was whether it would be possible to develop water-insoluble iodine-polymer complexes of medical relevance that have similar antimicrobial properties as iodophors and that are relatively simple to manufacture, with the iodine complexation step being integrated into existing process steps that are commonly used for processing of the base polymers, so that the resultant materials are relatively simple to manufacture and economically viable. Here, it is important to note that although there has been significant research on the fabrication of water-insoluble polymer-iodine (P-I) systems with biocidal activity, such systems are generally limited by two main issues: (i) the iodine source is PVP-I and/or (ii) the actual iodine complexation is not commercially viable. PVP-I is commercially available as a dry powder, of which the available iodine content represents about 10%. The use of PVP-I has two main disadvantages: (i) the addition of the desired amount of iodine will require a 9-fold addition of PVP to the system, and (ii) the PVP must not have a detrimental effect on the resultant product. PVP is a water-soluble, hydrophilic polymer, and substantial incorporation of said PVP product into a water-insoluble polymer matrix will have significant effects on the resultant product properties. One previous publication showed that the conjugation of PVP-I with cross-linked polystyrene resin could be used for bacterial decontamination of water, but in such cases, the PVP-I serves as a functional polymer rather than part of the fundamental engineering plastic matrix (14). There are also publications and reported research activities on the generation of water-insoluble polymer-iodine complexes in which the iodine is complexed with the matrix polymer via iodine solutions or vapor deposition. Though the resultant systems are shown to be biocidal, the lengthy post-iodine complexation process makes such processes cost-ineffective and difficult to manage from a good manufacturing practice (GMP) standpoint. One study generated 3D-printed polyvinyl alcohol-iodine stents that required 24 h of iodine vapor deposition, followed by 24 h of drying (15). In another study, iodinated polyurethane foams were produced by immersing said foams in an aqueous/organic solution of iodine for 24, 48, and 72 h to reach equilibrium (16), followed by subsequent drying of the foams. Though the resultant materials can offer unique antibacterial advantages and possess acceptable finished product properties, the lengthy postprocessing greatly compromises their commercial viability, both from cost and regulatory standpoints. It is also very difficult to develop robust processes for homogeneous iodine complexation (whether via iodine vapor deposition or iodine solutions) for water-insoluble polymers having various compositions and geometries.

Our present study reports the fabrication of iodine complexes of two medically relevant polymer families, specifically polyamides (PA) and polyurethanes (PU). The biocidal properties of the resultant fibers and foams are demonstrated for some of the most commonly encountered and clinically relevant microorganisms. Importantly, we also provide proof of principle that *in situ* generation of iodine-polymer complexes during regular production steps is feasible and greatly increases the practicality of the production systems. In brief, for the thermoplastic polyamide and polyurethane systems, iodine complexation was conducted in the “melt” via hot melt extrusion techniques. For the thermoset polyurethane systems, iodine complexation was conducted in the melt during the actual polymerization of monomers and additives. The synthesis of thermosets (i.e., cross-linked polymeric matrixes) of polyurethane-iodine foams was subsequently increased to industrial scale to further demonstrate their application potential. Altogether, our study shows that the described polymer-iodine complexes can be simply produced by integrating the complexation step into existing process steps already employed for the base polymer materials. Thus, both the equipment and time needed to produce the polymer-iodine systems are similar to those needed for the base polymer processing, making these systems uniquely positioned for commercial scale-up. The resultant polyamide-iodine (PA-I) and polyurethane-iodine (PU-I) complexes show strong bactericidal and fungicidal activity.

## RESULTS

### Production of polymer-iodine complexes.

PA-I and PU-I thermoplastic complexes were produced by direct extrusion of the base PA and PU polymers with a suitable iodine source in a twin-screw extruder. The extrusion conditions were dependent on the base PA and PU used and the ease of dissolving the iodine source into the molten PA matrix to generate the PA-I and PU-I complexes. The general extrusion temperatures were in the 150 to 240°C range, resulting in molten extrudates having stable melt viscosities and a transparent appearance. The final PA-I and PU-I cooled extrudates showed no surface defects, possessed undetectable vapor pressure from free iodine, and showed no iodine discoloration over extended time periods (i.e., months). Based on these observations, the incorporation and subsequent complexation of iodine with the PA and PU matrixes were deemed homogeneous and stable.

The PU-I thermosets were produced by first dissolving the suitable iodine source in one of the raw materials for PU fabrication, primarily the polyol or diol raw material. The PU reaction was then conducted as usual to produce the PU-I thermoset. In this case, homogeneous PU reactions in the melt will only occur if the reactive ingredients are mixed and reacted on the molecular level to initiate the polyurethane reaction. Thus, it can be concluded that the iodine used and complexed in these reactive PU systems must also be incorporated homogeneously on the molecular level throughout the final PU-I matrix. The resultant PU-I thermoset systems showed no iodine vapor pressure and no iodine discoloration over time. The only noticeable effect of the iodine on the PU polymerization reaction was the inhibition of the PU reaction. However, this could be overcome by slightly tweaking and optimizing the PU process by utilizing more reactive raw materials (i.e., aromatic isocyanates, primary alcohols, etc.) or by adding a catalyst. Iodine sources used for the complexation reactions included elemental iodine, and PVP-I (Table 1). The resultant extruded thermoplastic PA-I and PU-I fibers were directly tested for antimicrobial activity. The PU-I thermosets (foams) were allowed to cure and then cut into desired shapes for subsequent antimicrobial testing.

**TABLE 1:** Composition of the fibers and foams used in this study^*a*^

Sample	Composition (wt)	Total iodine (%)	Form	Microbial testing
1^*b*^	PA6 95%/PVP 5%	0	Fiber	Fig. 1 to 3
2	PA6 92.5%/PVP-I 5%/I 2.5%	3.0	Fiber	Fig. 1 to 3
3	PA6 90.5%/PVP-I 5%/I 4.5%	5.0	Fiber	Fig. 1 to 3
4	PA6 85.5%/PVP-I 5%/I 9.5%	10	Fiber	Fig. 1 to 3
6	PA6 95%/I 5%	5.0	Fiber	Fig. 1 to 3
7	PA6 93%/PVP 2%/I 5%	5.0	Fiber	Fig. 1
8	PA6 90%/PVP 5%/I 5%	5.0	Fiber	Fig. 1
9	PA6 85%/PVP 10%/I 5%	5.0	Fiber	Fig. 1
10	PA11 92.5/PVP-I 5%/I 2.5%	3.0	Fiber	Fig. 1
11	PA12 97%/I 3%	3.0	Fiber	Fig. 1
14	PA6 85%/PVP 5%/I 10%	10	Fiber	Fig. 1 to 3
15	PA6 90%/I 10%	10	Fiber	Fig. 1
16	PA Econyl 90.5%/PVP-I 5%/I 4.5%	5	Fiber	Fig. 1
17	PA6 Econyl 81%/PVP-I 10%/I 9%	10	Fiber	Fig. 1
18	PUR 95%/PVP-I 5%	0.5	Fiber	Fig. 1
19	PUR 92.5%/PVP-I 5%/I 2.5%	3.0	Fiber	Fig. 1
23^*b*^	PUR 100%	0	Fiber	Fig. 1
24	HMDI/glycerol/PEG400/I	0.5	Foam	Fig. 4
25^*b*^	HMDI/glycerol/PEG400	0	Foam	Fig. 4
26^*b*^	HMDI/glycerol/PEG400	0	Foam	Fig. 4
27^*b*^	HMDI/glycerol/PEG400/PVP K17	0	Foam	Fig. 4
28^*b*^	HMDI/glycerol/PEG400/water	0	Foam	Fig. 4
29	TDI/glycerol/PEG400/DABCO/I	2.0	Foam	Fig. 4 and 5
30	TDI/glycerol/PEG400/DABCO/I	0.5	Foam	Fig. 4
31	TDI/glycerol/PEG400/DABCO/PVP-I/I	2.0	Foam	Fig. 4 and 5
32	TDI/glycerol/PEG400/DABCO/I	1.0	Foam	Fig. 4
33	TDI/glycerol/PEG400/DABCO/PVP K17/I	1.0	Foam	Fig. 4
34	TDI/glycerol/PEG400/DABCO/I	3.0	Foam	Fig. 4 and 5
35	TDI/glycerol/PEG400/DABCO/PVP-I/I	1.0	Foam	Fig. 4
36	TDI/glycerol/PEG400/DABCO/I	0.5	Foam	Fig. 4
37	TDI/glycerol/PEG400/DABCO/I	1.0	Foam	Fig. 4
38^*b*^	HMDI/glycerol/PEG400	0	Foam	Fig. 4
39^*b*^	Novapore R110 commercial foam reticulated	0	Foam	Fig. 6 to 8
40	MDI/polyether polyol/polyester polyol nonreticulated	0.5	Foam	Fig. 6 and 7
41	MDI/polyether polyol/polyester polyol reticulated	0.8	Foam	Fig. 6 and 7
42	MDI/polyether polyol/polyester polyol nonreticulated	0.8	Foam	Fig. 6 and 7
43	MDI/polyether polyol/polyester polyol reticulated	0.5	Foam	Fig. 6 and 7
44	Commercial MDI/polyether polyol/polyester polyol reticulated	0.5	Foam	Fig. 8

a Samples 1 to 23 represent samples prepared via hot melt extrusion. Samples 24 to 44 represent samples prepared via PU polymerization reactions. Abbreviations and sources of the materials are detailed in Materials and Methods. The numbering sequence for the samples was based on the order of sample preparation rather than the order of biocidal testing. The omitted numbers relate to samples not included in the biocidal testing. PA6, polyamide 6; PVP, polyvinylpyrrolidone K30; PVP-I, polyvinylpyrrolidone-iodine; PA11, polyamide 11; PA12, polyamide 12; PUR, aliphatic polyether thermoplastic PU; I, elemental iodine; HMDI, hexamethylene diisocyanate; MDI, methylene diphenyl diisocyanate; PEG400, polyethylene glycol 400; PVP K17, polyvinylpyrrolidone K17; and DABCO, 1,4-diazabicyclo[2.2.2]octane.

b Control samples without iodine.

### Inhibitory effects of thermoplastic PA-I and PU-I fibers on the growth of various microorganisms: a qualitative evaluation.

**FIG 1:**
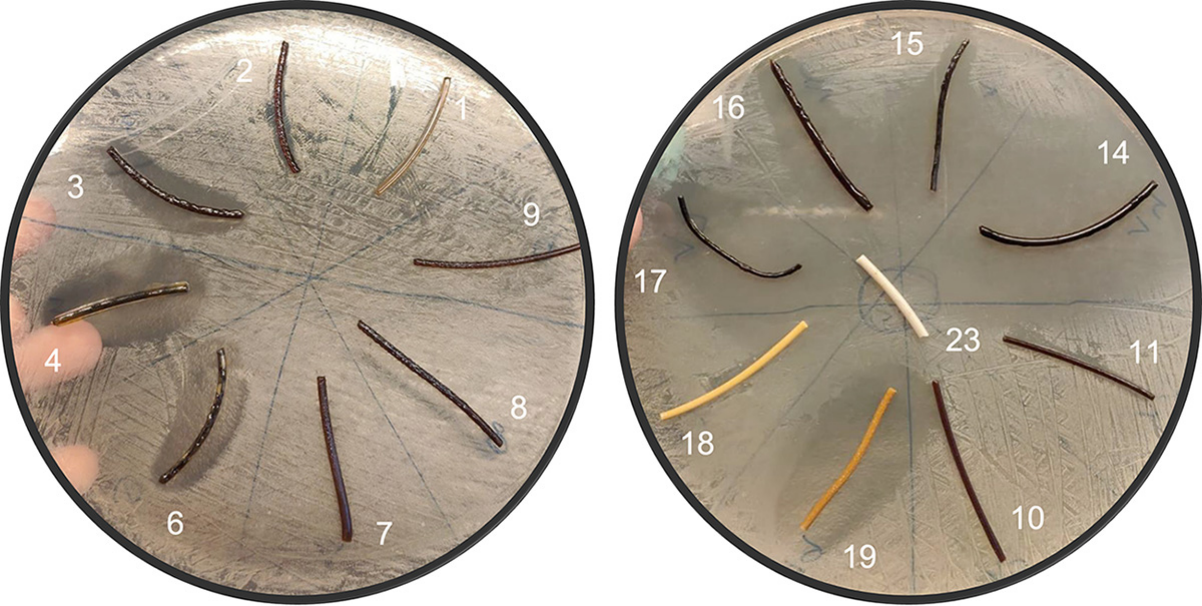
Inhibitory effects of PA- and PU-I thermoplastic fibers on growth of S. aureus. Materials 3, 4, 6, 14, 15, 16, 17, and 19 showed clear growth inhibition zones when placed on a plate with S. aureus SH1000 (MSSA). Material 1, which contained no iodine, served as a control.

To evaluate the potential growth inhibitory effects of P-I thermoplastic complexes on microorganisms, fibers composed of PA or PU extruded with PVP, PVP-I, and/or iodine at various concentrations (Table 1) were tested. Eight of the tested fibers (i.e., numbers 3, 4, 6, 14, 15, 16, 17, and 19) showed large clearing inhibition zones for S. aureus (Fig. 1). Fibers 2, 11, and 18 (containing lower iodine concentrations) showed more localized biocidal activity, with the clearing zones mainly apparent at the point of direct fiber contact. Fiber 1 served as the control sample, with no iodine addition, and accordingly, it showed no growth inhibition zone.

Based on the test results in Fig. 1, two main observations can be made. First, the size of the inhibition zones seems to be directionally related to the total iodine loading for like systems (see samples 2 to 4). Generally, higher iodine loading will lead to increased biocidal activity. However, second, there also seems to be a compositional effect of the polymer matrix on the biocidal activity, as indicated by samples 7 to 11. For these systems, there was minimal activity observed, though their overall iodine content was 3 to 5%.

**FIG 2:**
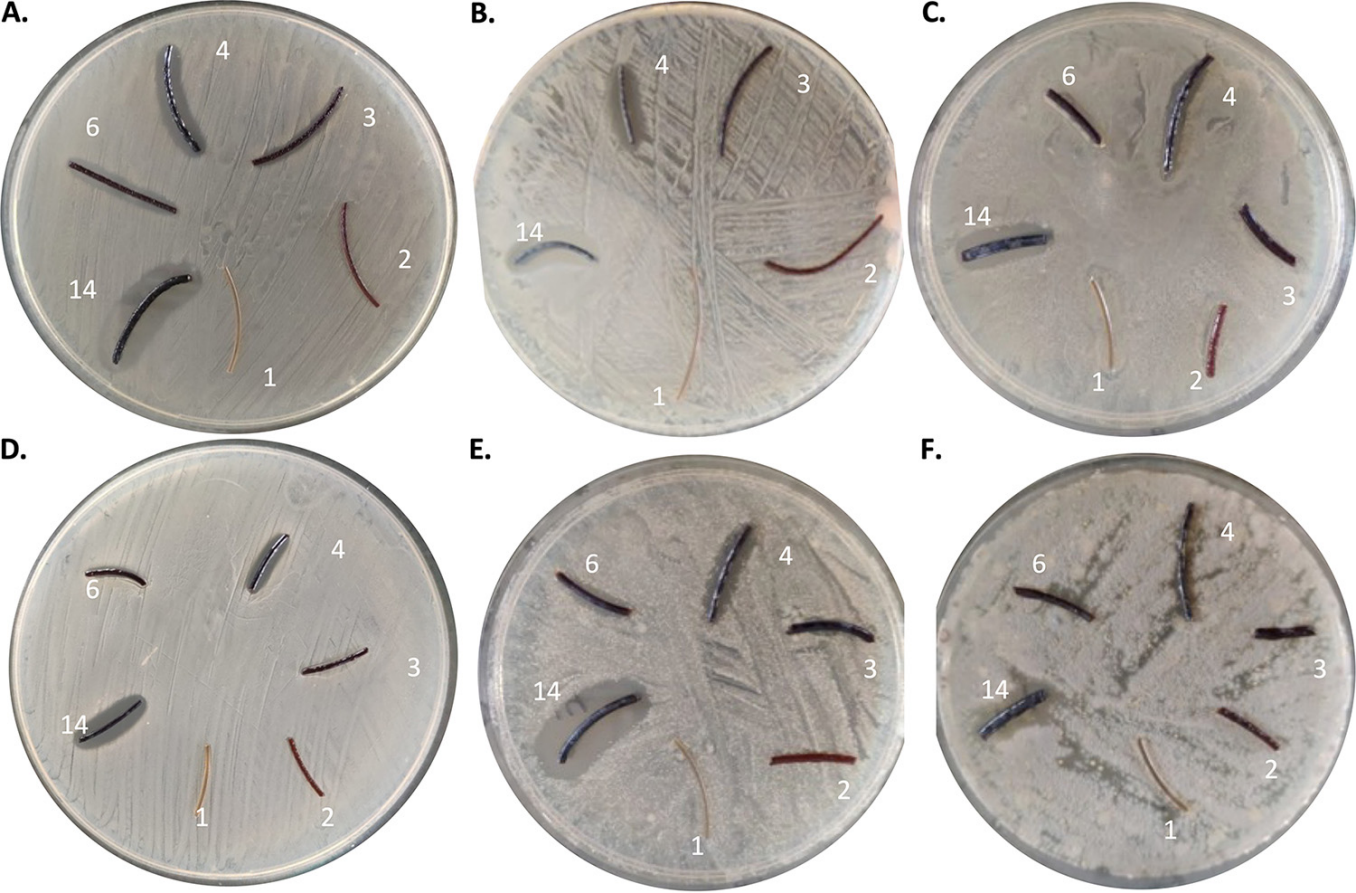
Inhibitory effects of PA-I fibers on various microorganisms: S. aureus SH1000 (MSSA) (A), S. aureus USA300 (MRSA) (B), S. pyogenes clinical isolate (C), S. epidermidis ATCC 35984 (D), C. albicans ATCC 10231 (E), and A. fumigatus 293 (F). For both S. aureus strains (A and B), S. pyogenes (C), and S. epidermidis (D), clear inhibition halos were observed with materials 4 and 14 compared to the iodine-free control material 1. For C. albicans (E), clear inhibition halos were observed for materials 4 and 14 and a less clear inhibition halo for material 6. For A. fumigatus (F), a clear inhibition halo was observed for material 14 and a smaller inhibition halo for material 4. Material 1, which contained no iodine, served as the control.

Fibers 2, 3, 4, 6, and 14 were selected to further evaluate the inhibitory effects on methicillin-susceptible Staphylococcus aureus (MSSA) SH1000, S. aureus USA300 (MRSA), Streptococcus pyogenes, Staphylococcus epidermidis ATCC 35984, Candida albicans ATCC 10231, and Aspergillus fumigatus 293 (Fig. 2). A clear growth inhibition of all investigated microorganisms was observed with fibers 4 and 14, containing 10% iodine loading. Though the P-I complexes showed activity against all indicated microorganisms, the biocidal effect was not always at the same level. Defined clearing zones were observed for fibers 3 and 6 at 5% iodine loading for S. aureus USA300 but not for S. pyogenes. It should be noted that the plating studies utilized a high concentration of microorganisms. Such contamination concentrations would never likely occur in an actual health care setting. Expected contamination would occur at significantly lower concentrations and specific to the medical device surface, where significantly lower levels of iodine loading will probably suffice to reduce the infection risk.

### Inhibitory effects of PA-I fibers following sterilization.

**FIG 3:**
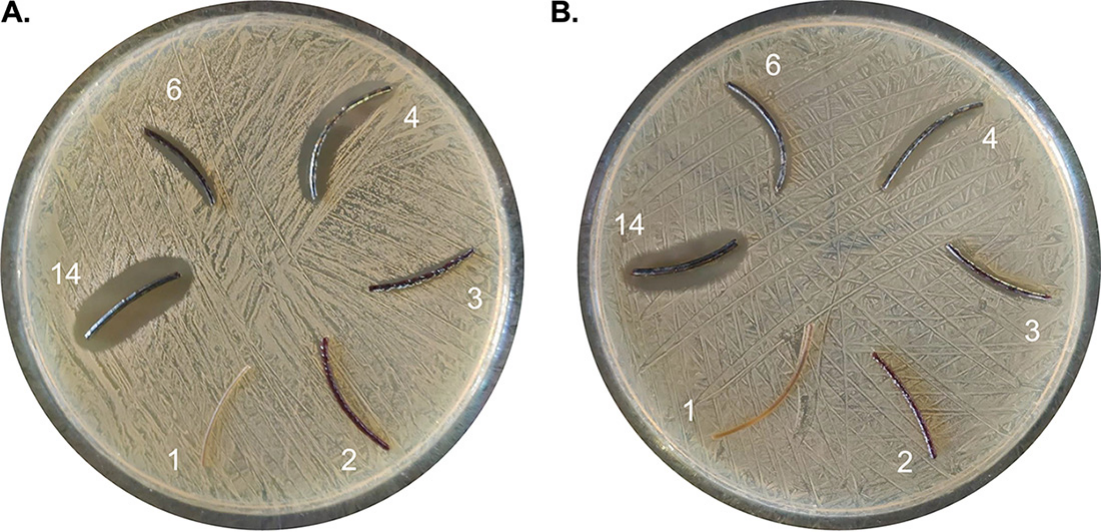
Growth inhibitory effects of PA-I fibers following sterilization on S. aureus SH1000 (MSSA) (A) and S. aureus USA300 (MRSA) (B). For both S. aureus strains, a clear inhibition halo was observed for the sterilized materials, samples 4, 6, and 14, compared to the sterilized iodine-free control material, sample 1.

To evaluate the possibilities for reuse of the different materials, a potential decrease of the inhibitory effect following sterilization was tested. For both strains of S. aureus (MRSA and MSSA), the formation of halos reflecting growth inhibition was still observed upon autoclaving (121°C, 15 lb/in^2^, 15 min) (Fig. 3). This observation also strongly supports the view that antibacterial activity of the iodine-containing materials will be observed over an extended time period, which is an important factor for medical devices that need to remain in contact with the body for extended periods of time (e.g., wound dressings, urinary catheters, drains, etc.).

### Inhibitory effects of PU-I foams on the growth of various microorganisms.

**FIG 4:**
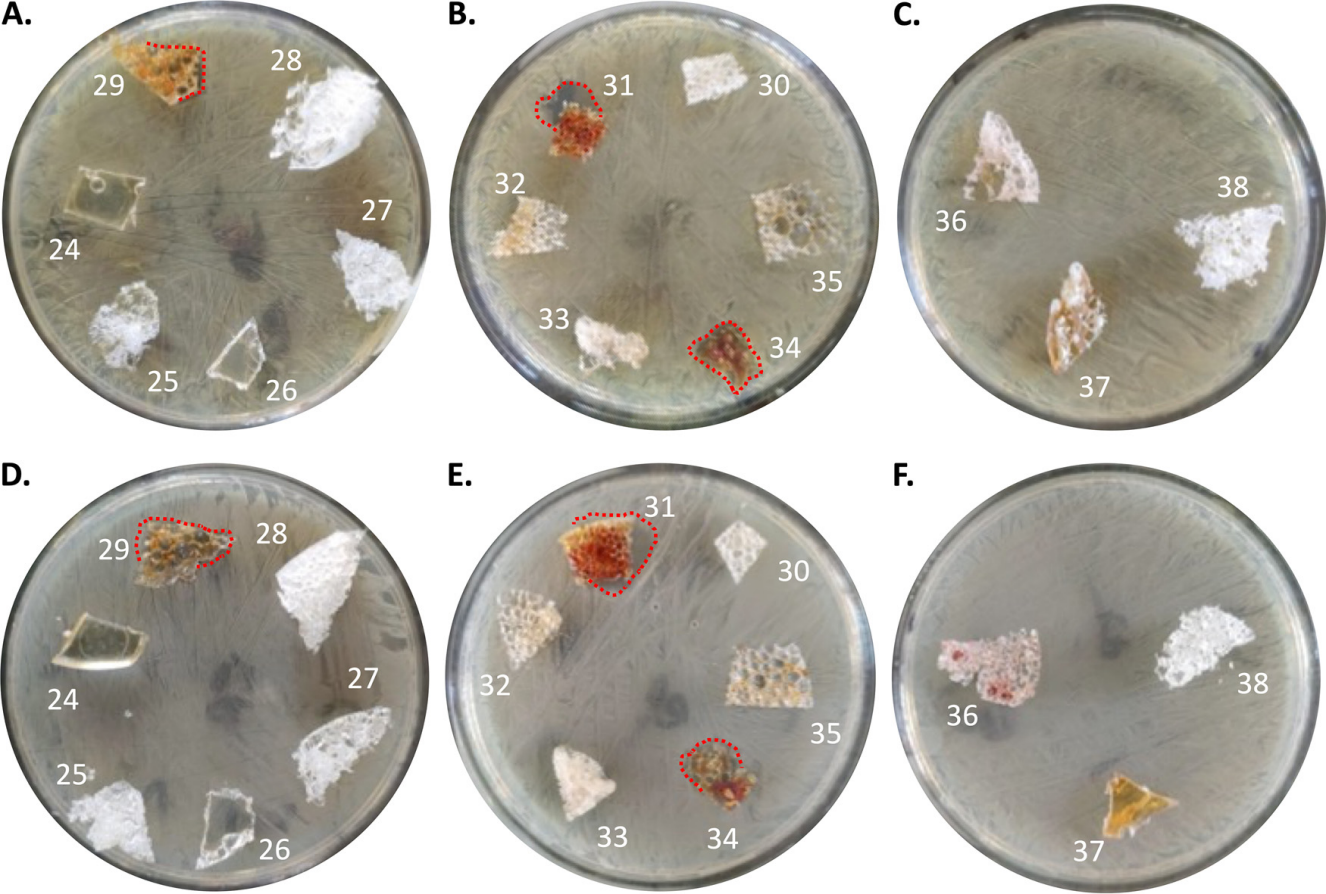
Growth inhibitory effects of laboratory PU-I foams on the bacterial growth of an S. aureus clinical isolate (A to C) and S. epidermidis ATCC 35984 (D to F). For both S. aureus and S. epidermidis, a clear inhibition halo was observed with materials 31 and 34. Material 29 caused a small growth inhibition halo. Inhibition halos are marked with a red dotted line. Materials 25 to 28 and 38, which contained no iodine, served as controls.

**FIG 5:**
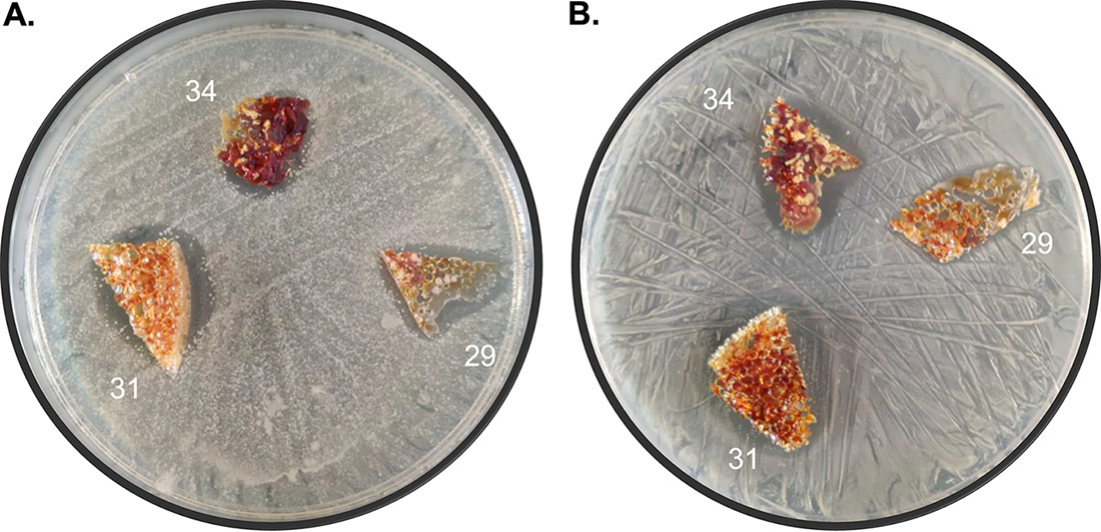
Growth inhibitory effects of selected laboratory PU-I foams on the yeast C. albicans ATCC 10231 (A) and an S. pyogenes clinical isolate (B). For both microorganisms, clear inhibition halos were observed with all tested materials (samples 29, 31, and 34).

In addition to the thermoplastic PU systems, research was also initiated to evaluate the biocidal activity of thermoset PU-I foam systems. The PU-I foams were prepared as outlined in Materials and Methods. The *in situ* generation of the iodine complex during the actual polymerization reaction is a unique observation for this study. The *in situ* process to produce PU-I thermosets seems to be generic to polyurethane systems in general, and thus, it is expected that a broad range of PU-I systems can be produced simply and economically. The inhibitory effects on microbial growth of lab-produced foams composed of different formulations (Table 1) were first tested on a clinical S. aureus isolate and the S. epidermidis type strain ATCC 35984. Foams 29, 31, and 34 showed clear growth inhibition halos (Fig. 4), and therefore, they were further evaluated for inhibition of C. albicans ATCC 10231 and a clinical strain of S. pyogenes that had been obtained after sonication of an implanted catheter (Fig. 5).

**FIG 6:**
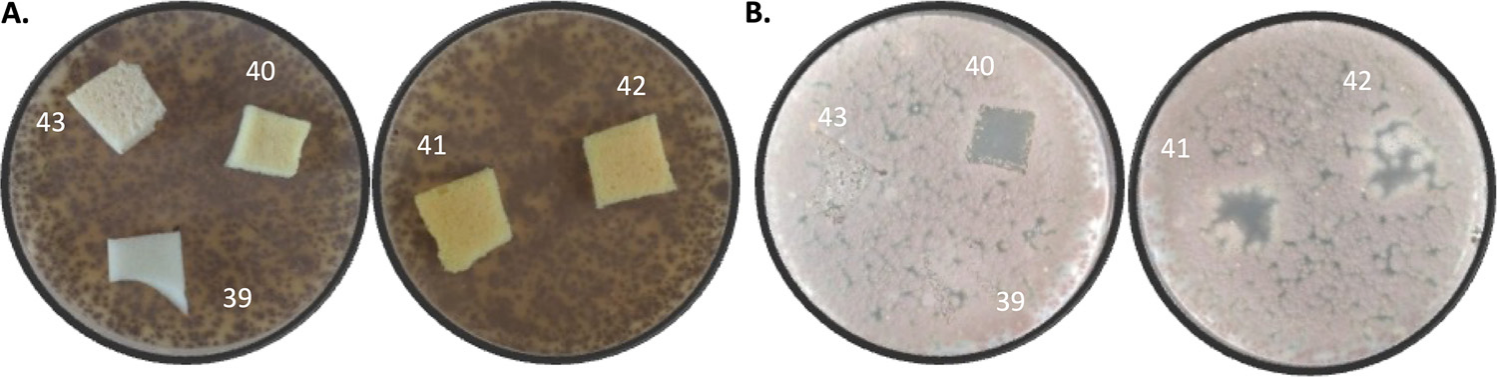
Growth inhibitory effects of development foams on A. fumigatus. (A) Second-generation foams 39 to 43 on top of Mueller-Hinton agar (MHA) plates. (B) Inhibition halos from foams 39 to 43. Clear halos were observed with foams 40, 41, and 42, compared to the iodine-free control (material 39).

Based on the positive results from various lab-produced PU-I foams, new development foams, both reticulated and nonreticulated, were produced at pilot-relevant scale (samples 39 to 43; Table 1) and further evaluated against A. fumigatus (Fig. 6). Due to the lower iodine loading in these foams, the fungal inhibition halo under the foams was evaluated to determine activity. Noteworthy is the observation that the fungicidal activity reflected by the clearing zones seemed less related to the iodine levels and more to the actual contact of the foams with A. fumigatus on the growth medium. In this respect, it is relevant that the light and buoyant PU-I foams do not all have the same contact points and that the foams are either reticulated (open cell) or nonreticulated (closed cell). Accordingly, the capillary effects of liquid can have differential effects on the observed inhibitory zones. This observation implies that the compositional makeup of the PU foam with respect to wetting can also have indirect effects on the biocidal activity. This parameter should be considered in medical device design.

### Inhibitory effect of PU-I foams on saliva samples from healthy volunteers.

**FIG 7:**
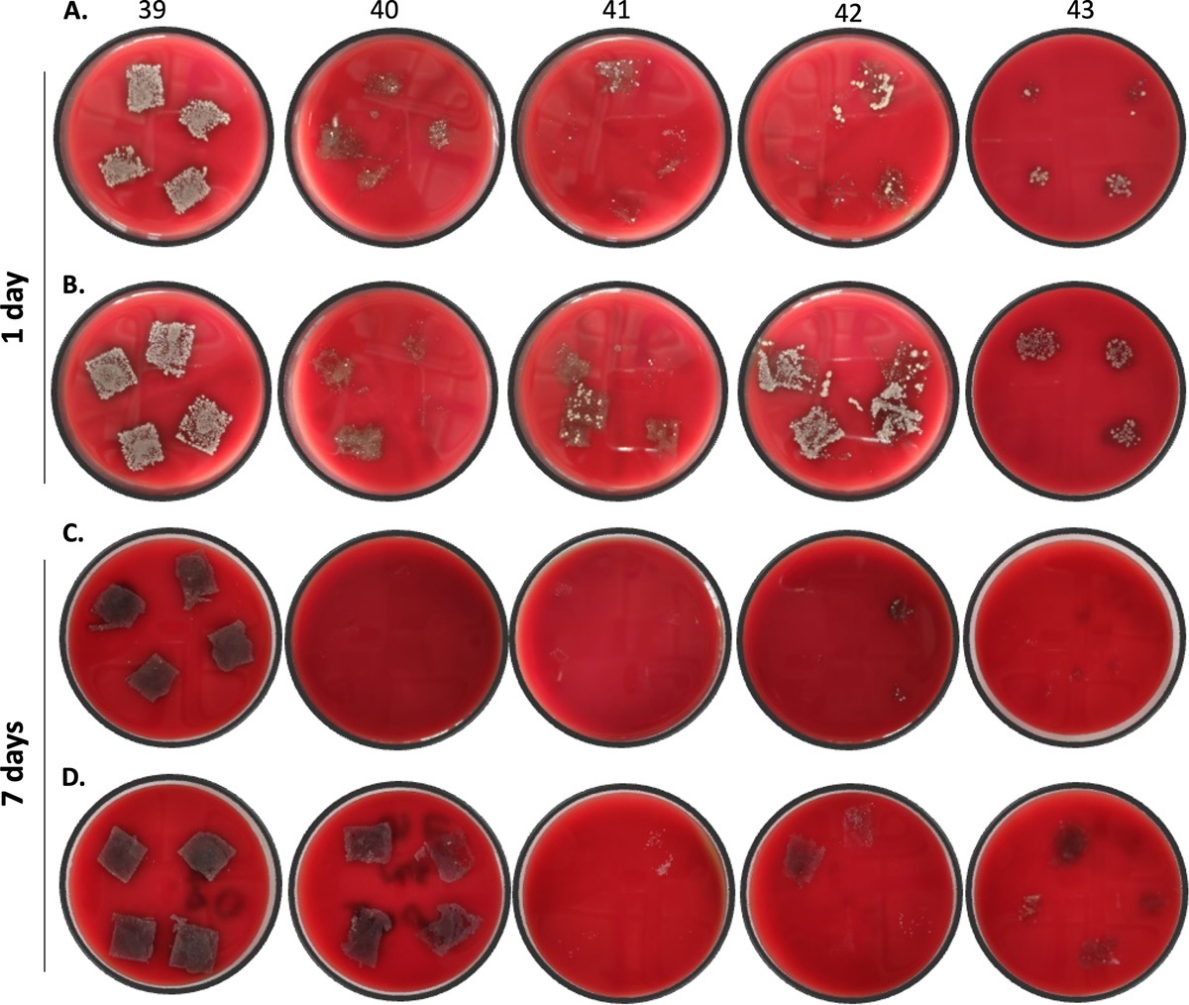
Microbial growth inhibition following preincubation of various foams with pooled saliva for 1 (A and B) or 7 (C and D) days. The microbial load was visualized by pressing foams 39 to 43 for 10 s onto a blood agar plate and subsequently incubating them overnight. For panels B and D, the foams were autoclaved prior to the experiment. All foams showed a clear inhibition of microbial growth compared to the iodine-free control (material 39).

Due to the current great need for face masks, the developed foams were further evaluated for inhibition of oral microorganisms. Freshly prepared and identical autoclaved foams (samples 39 to 43; Table 1) were incubated with pooled saliva samples from healthy volunteers for 1 or 7 days at room temperature. Subsequently, the foams were replica plated by pressing them onto blood agar (BA) plates. Foams 40 to 43 showed a clear decrease in microbial load compared to the iodine-free control foam (39) after a 1-day incubation of the saliva-inoculated material (Fig. 7A and B). After 7 days of incubation, the inhibitory effect was nearly 100% for the nonautoclaved foams (Fig. 7C and D). Upon autoclaving, the foams retained their biocidal activity to various extents.

### Inhibitory effect of commercially produced PU-I face masks on microbial growth.

**FIG 8:**
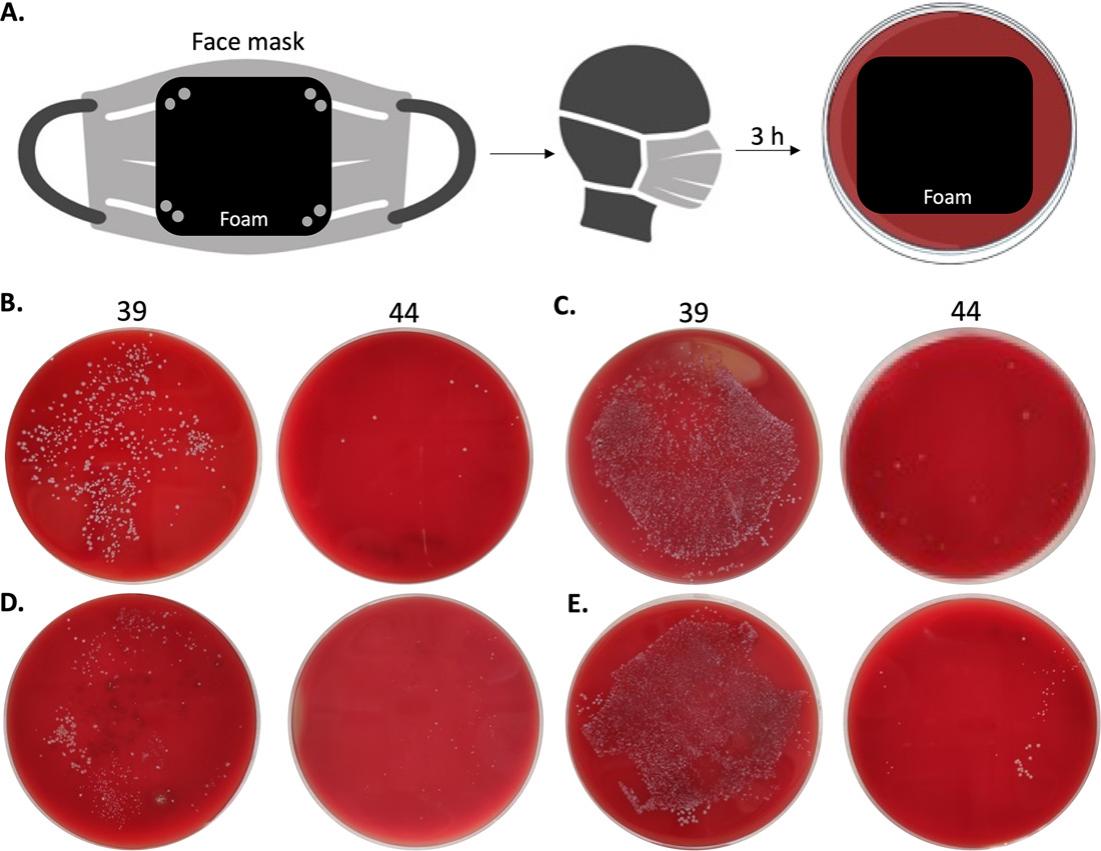
Microbial growth-inhibiting effects of PU-I foam face masks. (A) Schematic representation of the methodology (created using BioRender). (B to E) Face masks made with foams 39 (control) or 44 (0.5% iodine) were worn for 3 h by a healthy volunteer and replica plated for 10 s on top of a BA plate (B) or kept on top of a BA plate (C) during a 24-h incubation at 37°C. (D and E) Results represent the same experiment as conducted in panels B and C, respectively, except that the face masks were washed 1× at 60°C prior to usage.

Based on the above positive results, a commercial batch of reticulated foam 44 was produced at a batch size of 1,500 kg in order to show that the technology was scalable and commercially viable. The resultant high-quality foam was tested as a potential component for the development of face masks with microbial inhibitory properties. These face masks were worn for 3 h by healthy volunteers. Subsequently, the side of the foam that was in contact with the volunteer’s face was pressed onto a blood agar plate to assess the bacterial load. As shown in Fig. 8, the microbial load of face masks incorporating foam 44 was significantly decreased in comparison with the control, foam 39 (reticulated commercial foam with no biocidal activity).

## DISCUSSION

Considering the impact of HAIs in many clinical settings, developing medical devices with inherent properties against bacterial contamination and biofilm formation is of great importance. Many studies are focused on the evaluation of coating agents such as polymer coatings, antimicrobial coatings, and nanostructured coatings to antagonize the first adherence of microorganisms and thus inhibit or slow down biofilm formation (16). From such studies, the use of antiseptic-based coatings was shown to be more effective compared to the use of antibiotic-based coatings (16, 17). Moreover, the local delivery of antiseptics was successful when implemented in coatings for catheter-based applications (18, 19). The use of metal salts and metal nanoparticles has also been implemented in many medical devices to control infections, with silver systems having significant applications in medical device development (20, 21). However, there remains a growing interest in finding new antiseptic systems that potentially better manage safety concerns (e.g., reduced leaching of the antiseptic from the medical device) or better deal with new regulatory protocols (21, 22).

In the present study, we show that the excellent antiseptic properties of water-soluble povidone-iodine can be similarly realized in water-insoluble engineering plastics, specifically PA- and PU-iodine complexes. These inherently antibacterial complexes can be manufactured cost-effectively via known manufacturing techniques. In addition, the use of elemental iodine itself in producing the polymer-iodine complex *in situ* offers more flexibility with respect to material development. It is expected that such process conditions will make these iodine complexed materials more commercially relevant for the fabrication of antibacterial/antiviral articles for application in health care, consumer care, and industry.

**FIG 9:**
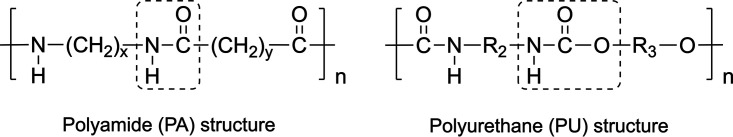
Structures of PA and PU.

Though more research must be conducted, the PA- and PU-iodine complexes described here are thought to be driven by hydrogen bonding and charge complex interactions between the iodine and amide and urethane groups of the PA and PU (Fig. 9), respectively. Similar interactions have been detailed for other polymer-iodine structures elsewhere (4).

The PA- and PU-iodine complex materials show no vapor pressure and/or iodine discoloration over time, resulting in extended activity against a broad range of pathogens. For polymeric systems that show no complex formation with iodine (for example, polypropylene), the resultant polymer-iodine blend obtained via hot melt extrusion was not stable, resulting in an iodine vapor pressure that immediately caused discoloration of adjacent materials. Our results thus provide proof of principle that PA-I and PU-I complexes can be produced that are commercially viable and show broad biocidal activity. The antiseptic attributes of PVP-I were successfully integrated into two commonly used biomaterial families, polyamides and polyurethanes, by the complexation with iodine. We also showed that the level of iodine concentration or the incorporation of other ingredients in the systems can have a significant effect on the growth inhibitory zones in our plate assays, suggesting that the polymer-iodine systems can be further customized to meet specific requirements for a particular medical need. Moreover, we showed that the biocidal properties of the polymer-iodine complexes included in this study are largely maintained upon sterilization, which strongly supports the view that the materials can be reused or will maintain their biocidal activity for an extended period of use. Thus, these polymers have the extra advantage of a low environmental footprint, since they can be recycled or allowed to remain in use for longer periods of time.

In the case of PU-I foams, we show that the technology is readily scalable and that commercial production of both nonreticulated (closed-cell) and reticulated (open-cell) foams with biocidal activity is viable. The present research shows that the biocidal activity observed in the nonreticulated PU-I foam is maintained after reticulation. Finally, we developed a prototype face mask with a commercially produced PU-I reticulated foam that shows strong biocidal activity against oral bacterial flora. Reticulated PU foams are presently used as air-filtering media in a number of applications (e.g., air conditioners, community masks, etc.). The production of similar filtering media using reticulated PU-I foams could be beneficial to control the growth and spread of pathogens, especially for systems used in high-moisture applications, such as air conditioning and masks.

### Conclusion.

In conclusion, the beneficial antimicrobial attributes of water-soluble PVP-I can be similarly realized in PA-I and PU-I materials. The production process for the PA-I and PU-I systems is simple, and the iodine complexation step can be integrated into existing process steps, which greatly improves their commercial viability. Both PA and PU materials show excellent biocompatibility and thus are used for the fabrication of a broad range of medical devices. Though this initial proof-of-concept study focuses mainly on the biocidal properties and potential commercial viability of PA-I and PU-I complexed materials, the ability to introduce desirable biocidal attributes through the complexation of iodine should be a strong justification for further development of these materials.

## MATERIALS AND METHODS

### Preparation of the fibers and foams.

Thermoplastic PA and PU systems (samples 1 to 23) were prepared via hot melt extrusion, using a Thermo Prism EuroLab 16 twin screw extruder having a 16-mm screw diameter and a 25-cm barrel length. The extruding barrel had five heating zones that were set in the range of 150 to 240°C, depending on the system being compounded. The rotation speed of the screws was fixed at 400 rpm. The powders/solids were initially dry mixed and then fed into the extruder. The extruded filament was water cooled and cut to length to produce the fibers. The following materials were used: polyamide 6 (PA6; Lanxess Durethan 29), polyamide 6 (PA6 Econyl; Aquafil ECONYL 6), polyvinylpyrrolidone K30 (PVP; Boai NKY Pharmaceuticals Ltd. PolyViscol K30), polyvinylpyrrolidone-iodine (PVP-I; Boai NKY Pharmaceuticals Ltd. KoVidone-I), polyamide 11 (PA11; Sigma Aldrich/Merck), polyamide 12 (PA12; Sigma Aldrich/Merck), aliphatic polyether thermoplastic PU (PUR; Lubrizol TecoFlex EG-93A-B30), and elemental iodine 99.8% (Sigma Aldrich/Merck).

Thermoset PU systems (samples 24 to 38) were prepared by the reaction of an isocyanate with a polyethylene glycol/glycerol polyol blend, with or without an iodine source. If an iodine source was used in the reaction, the iodine was initially dissolved in the polyol blend prior to conducting the polyurethane reaction. Polyurethane reactions including an iodine source required the addition of a catalyst (DABCO) to accelerate the reaction. The polyurethane reaction was conducted by reacting the isocyanate with the polyol source at room temperature until homogeneous, and the reaction mixture was then poured into molds and placed in a 60°C oven for 2 h. The following materials were used: hexamethylene diisocyanate (HMDI; Sigma Aldrich/Merck), toluene diisocyanate (TDI; Sigma Aldrich/Merck), polyethylene glycol 400 (PEG400; Sigma Aldrich/Merck), glycerol (Sigma Aldrich/Merck), polyvinylpyrrolidone K17 (PVP K17), and 1,4-diazabicyclo[2.2.2]octane (DABCO; Sigma Aldrich/Merck).

Thermoset PU systems (samples 39 to 44) were kindly prepared by Caligen Europe BV. The PU systems were based on commercial processes and raw materials and supplied in their corresponding nonreticulated and reticulated forms. The reticulation process was conducted by combustion. Sample 39 was a commercial reticulated foam containing no iodine (i.e., the control). Samples 40 to 43 were lab samples based on MDI (methylene diphenyl diisocyanate)/polyether polyol/polyester polyol/iodine foams that were produced at the kilogram scale. Sample 44 was a reticulated foam based on MDI/polyether polyol/polyester polyol/iodine commercially produced at a batch size of 1,500 kg. The resultant commercial PU foam bricks were then reticulated and finally peeled to give the final foam, having desired thicknesses of 1.5 to 3 mm.

All subsequent biological experiments with the developed PA- and PU-based materials were replicated at least twice.

### Inhibition effect of PA/PU-I fibers/foams on microbial growth.

Methicillin-susceptible Staphylococcus aureus (MSSA) SH1000, methicillin-resistant S. aureus (MRSA) USA300, a clinical Streptococcus pyogenes isolate, S. epidermidis ATCC 35984, Candida albicans ATCC 10231, and Aspergillus fumigatus 293 were grown in tryptic soy broth (TSB) overnight at 37°C. Aliquots (100 μL) of the overnight cultures were diluted to an optical density at 600 nm (OD_600_) of 0.1 and subsequently plated onto 100 mm Mueller-Hinton agar (MHA) plates. Fibers or foams with different concentrations of nylon, PVP, and iodine were placed on top of the plates, and the plates were then incubated at 37°C. Growth inhibition was inspected upon 24 and 48 h of incubation.

### Microbial growth inhibition by PA-I fibers following sterilization.

To evaluate a possible decrease in the microbial growth inhibitory efficacy of the investigated materials after standard sterilization by autoclaving, the fibers were washed once with 70% ethanol, rinsed with distilled H_2_O, dried, and autoclaved at 121°C (15 min, 15 lb/in^2^).

### Growth-inhibiting effects of PU-I foams on microbe-containing saliva samples from healthy volunteers.

To evaluate the effects of PVP-I on oral microorganisms, saliva samples from healthy volunteers were pooled and mixed to homogeneity using a vortex mixer. Subsequently, 1-mL aliquots of the pooled saliva were incubated with various foams (samples 39 to 43; Table 1). After 1 or 7 days of incubation at room temperature, the foams were replica plated by gently pressing them onto blood agar plates (BA) for 10 s. Subsequently, the plates were incubated for 24 h at 37°C. To ensure that all the microorganisms present in the foams were detected, the materials incubated with saliva for 7 days were also plated and kept on top of the BA plates during incubation for 24 h at 37°C. Evaluation of the growth inhibitory effects of 1× autoclaved foams was carried out in parallel.

### Microbial growth-inhibiting effects of PU-I foam mouth masks.

Prototype face masks were developed with reticulated commercial PU foams containing iodine (sample 44) and without iodine (sample 39) to evaluate the activity of these foam materials on microorganisms commonly transmitted from the face and breath to face masks. Mouth masks were worn for 3 h by volunteers. Afterwards, the inside of the mask was gently pressed for 10 s onto BA plates. The plates were subsequently incubated for 24 h at 37°C. After pressing the inside and outside of the mask onto BA plates, a piece of the mask was cut and incubated on top of a BA plate at 37°C for 24 h.

### Ethical approval.

Studies with healthy volunteers wearing PU-I foam face masks were performed based on written informed consent with approval of the Medical Ethics Review Board of the University Medical Center Groningen (UMCG; approval number Metc2012-375) and with adherence to the Helsinki Guidelines.

### Data availability.

All data and materials are available upon request.
